# Dietary Intake and Health Status of Elderly Patients With Type 2 Diabetes Mellitus: Cross-sectional Study Using a Mobile App in Primary Care

**DOI:** 10.2196/27454

**Published:** 2021-08-27

**Authors:** Joane Diomara Coleone, Ericles Andrei Bellei, Mateus Klein Roman, Vanessa Ramos Kirsten, Ana Carolina Bertoletti De Marchi

**Affiliations:** 1 School of Physical Education and Physiotherapy University of Passo Fundo Passo Fundo, RS Brazil; 2 Institute of Exact Sciences and Geosciences University of Passo Fundo Passo Fundo, RS Brazil; 3 Department of Foods and Nutrition Federal University of Santa Maria Palmeira das Missões, RS Brazil; 4 Graduate Program in Gerontology Federal University of Santa Maria Santa Maria, RS Brazil

**Keywords:** eating, mobile applications, primary health care, aged, type 2 diabetes mellitus

## Abstract

**Background:**

Healthy dietary intake reduces the risk of complications of diabetes mellitus. Using assessment methods helps to understand these circumstances, and an electronic application may optimize this practice.

**Objective:**

In this study, we aimed to (1) assess the dietary intake and health status of elderly patients with type 2 diabetes mellitus (T2DM) in primary care, (2) use a mobile app as a tool for data collection and analysis in the context of primary care, and (3) verify the perceptions of multidisciplinary health professionals regarding app use.

**Methods:**

First, we developed a mobile app comprised of the questions of the Food and Nutrition Surveillance System (SISVAN) of Brazil, which includes a food frequency questionnaire of food categories with a recall of the previous 7 days. Thereafter, we used the app to collect data on the health status and dietary intake of 154 participants, aged 60-96 years, diagnosed with T2DM, and under treatment in primary care centers in the northern region of Rio Grande do Sul, Brazil. We also collected participants’ demographic, anthropometric, biochemical, and lifestyle variables. The associations between dietary intake and other variables were tested using chi-square tests with a 5% significance level. Regarding the app, we assessed usability and acceptance with 20 health professionals.

**Results:**

Between August 2018 and December 2018, participants had an intake in line with recommended guidelines for raw salads (57.1%), fruits (76.6%), milk products (68.2%), fried foods (72.7%), savory biscuits (60.4%), cookies or sweets (72.1%), and sugary drinks (92.9%) Meanwhile, the consumption of beans (59.7%), pulses and cooked vegetables (73.4%), and processed meat products (59.7%) was not in line with the guidelines. There were statistically significant differences in meeting the recommended guidelines among participants of different genders (*P*=.006 and *P*=.035 for the intake of fried foods and sugary drinks, respectively), place of residence (*P*=.034 for the intake of cookies and sweets), family history of diabetes (*P*<.001 for the intake of beans), physical activity engagement (*P*=.003 for the intake fresh fruits), history of smoking (*P*=.001 for the intake of raw salads), and presence of coronary disease (*P*=.050 for the intake of pulses and cooked vegetables). The assessment of usability resulted in a mean score of 71.75 points. Similarly, the assessment of the 15 acceptance questions revealed high scores, and the qualitative questions revealed positive perceptions.

**Conclusions:**

We identified that most participants complied with recommended intake guidelines for 7 of 10 categories in the SISVAN guidelines. However, most participants were overweight and had nutritional and clinical disorders, which justifies further investigations in this population. The app was well-rated by health professionals and considered a useful and promising tool for collecting and analyzing data in primary care settings.

## Introduction

An aging population, the prevalence of obesity, sedentary lifestyles, and urbanization processes have been contributing to the increase in type 2 diabetes mellitus (T2DM) worldwide [1]. In public health, especially in developing countries such as Brazil, T2DM results in high economic and social costs for its treatment and care, due to the association with several complications [2]. A healthy dietary intake reduces the risk of complications to maintain acceptable health standards and functional capacity [3]. However, patients with T2DM, particularly the elderly, have difficulty adhering to a healthy diet, as they may perceive diet plans as prohibitive, restrictive, and challenging [4]. In these circumstances, understanding dietary habits requires appropriate assessment tools.

A systematic review [5] revealed that the main method for assessing dietary intake in public health settings for elderly patients with T2DM can be classified as a food frequency questionnaire. However, the application of the questionnaire is paper-based in most cases, which is time-consuming. New technologies offer great opportunities to fully assess the intake of foods and nutrients of large populations at relatively low cost and in real time [6]. These tools have fast access, full-time availability, and potential access through mobile apps, showing practicality for hospitals, clinics, and outpatient clinics [7]. In Brazil’s public health system, primary care is the main strategy of surveillance, which also aims to provide comprehensive health care, specialized services, and hospital care, as well as health promotion and disease prevention activities [8]. In these settings, although still not widespread, the use of technology is promising [9,10], in particular to facilitate the work of multidisciplinary health professionals. Therefore, initiatives to introduce digital tools are relevant to test possibilities and improve primary care in Brazil.

In this study, we aimed to (1) assess the dietary intake and health status of elderly patients with T2DM in primary care, (2) use a mobile app as a tool for data collection and analysis in the context of primary care, and (3) document the perceptions of multidisciplinary health professionals regarding app use.

## Methods

### Overview

We considered the sequences and processes from several studies in the literature involving software applications [11-13] to select, develop, and apply an assessment tool complying with the DIET@NET partnership guidelines [14]. We performed a cross-sectional study between August 2018 and December 2018 in public primary health centers in 4 small towns in the northern region of Brazil's Rio Grande do Sul, namely Estação, Erebango, Getúlio Vargas, and Ipiranga do Sul, which had 27,079 inhabitants in the last official census. This study was conducted according to the guidelines laid down in the Declaration of Helsinki, and all procedures involving research study participants were approved by the ethics committee of the University of Passo Fundo under opinion number 2660304. Written informed consent was obtained from all participants. According to national regulations, participants were not compensated.

### Sampling, Subjects, and Recruitment

For the sample calculation, we considered the total population of elderly patients who were diagnosed with T2DM by the public health control from the cities investigated and attended a primary care center (N=257), with an expectation of a dietary intake 50% in line with the Food and Nutrition Surveillance System (SISVAN) of Brazil guidelines, acceptable error of 5%, and a confidence level of 95%, resulting in a target sample size of 154 individuals. The inclusion criteria were 60 years of age or older; T2DM diagnosis, either by self-report or confirmed by examinations in the patient history (fasting glycemia or glycated hemoglobin [HbA_1c_]); and cognitive ability confirmed by the Mini-Mental State Examination [15]. Through phone calls, we randomly called participants to attend the primary health center for an interview. We invited participants until we reached the required sample. All participants signed an informed consent form.

### Development of a Mobile App for Dietary Assessment

Initially, we performed a systematic mapping to investigate the different methods for assessing the dietary intake of adults and the elderly [5]. We observed that the main assessment method was a food frequency questionnaire. However, the questionnaires are usually applied manually. To optimize time and resources, we wanted to use an electronic method for collecting and processing the questionnaires.

In this study, we used the Dietary Intake Form proposed by SISVAN [16], which is an initiative from the Brazilian Ministry of Health. The form is a food frequency questionnaire with a recall period of the previous 7 days and is comprised of 10 categories, which relate to a healthy diet (eg, daily intake of beans, fresh fruits, vegetables, and milk) or to nonrecommended practices (eg, frequent intake of fried foods, processed meat products, cookies, sweets, and soft drinks). SISVAN guidelines recommend an intake of 7 serves per week of raw salad, pulses and cooked vegetables, fresh fruit, and milk or yogurt; 5 or more serves per week of beans; and 0-1 serves per week of fried foods, processed meat products, savory biscuits, cookies or sweets, and sugary drinks.

Previously, a mobile app addressing SISVAN had been developed for use with inpatients [7]. Hence, we developed a new version, with improved functionalities adapted to primary care outpatients, called Diabetes Food Control 2. The app now includes questions on demographic and economic characteristics (eg, marital status, self-reported skin color, education, income, and occupation), clinical and nutritional history, time from diagnosis, family history of diabetes, medication intake, presence of comorbidities, nutritional monitoring, lifestyle, physical activity, smoking, and alcohol consumption. The Brazilian Institute of Industrial Property granted us a software registration certificate under number BR5120190010540.

**Figure 1:**
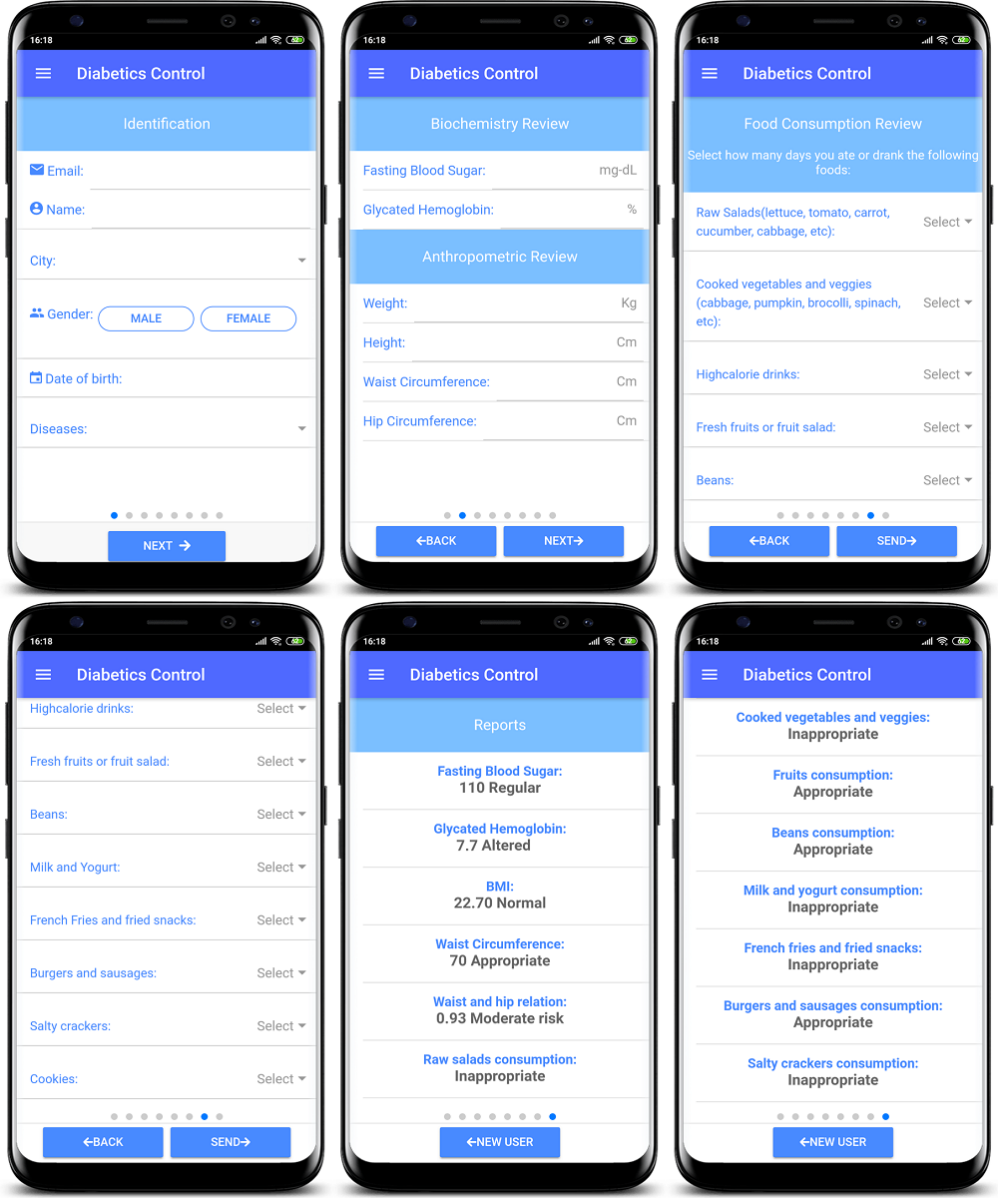
Screenshots of the Diabetes Food Control 2 mobile app showing patient data collection forms and feedback on the data collected.

The app (Figure 1) was designed so that health care professionals could use it in primary health care centers to assess the dietary intake and health status of outpatients with T2DM, addressing the recommendations of Brazil's Ministry of Health [16]. Using the app, we intended to optimize data collection and make the usage of apps a more consistent practice in primary care settings. The app's report provides straightforward information on whether the patient meets the SISVAN criteria related to food intake and anthropometric measurements. It also provides personalized feedback for patients in the form of a report they can receive by email.

### Procedures

We collected all data with the SISVAN questionnaire using the Diabetes Food Control 2 app. In the public primary health centers selected for the study, the same dietitian researcher interviewed each participant to simultaneously fill out the questionnaire on the app. The dietitian also collected demographic and economic data such as marital status, self-reported skin color, level of education, income, and occupation. The participants followed the use of the app during the entire interview and interacted by filling out their demographic information.

To evaluate nutritional status, we analyzed body weight (kg), height (cm), and waist and hip circumferences (cm). For stature measurement, we used a 200-cm stadiometer with a scale of 0.2 centimeters. To assess weight, we used a Welmy digital scale with a capacity of 150 kg. We took all measurements according to the recommendations of the Brazilian Ministry of Health [17]. Using the measured height and weight, the app calculated the BMI and the classification of nutritional status according to the guidelines of the Pan American Health Organization [18].

The dietitian assessed waist circumference with a measuring tape at the smallest curvature between the ribs and the iliac crest, without compressing the skin. For waist circumference, the categories were low risk (men <94 cm; women <80 cm), high risk (men ≥94 cm; women ≥80 cm), and very high risk (men ≥102 cm; women ≥88 cm). We measured hip circumference in centimeters in a horizontal plane at the region of the greatest gluteal protuberance. We calculated the waist-to-hip ratio according to the cutoff points recommended by the World Health Organization [19]. For waist-to-hip ratio, the categories were low risk (men <0.9; women <0.8), moderate risk (men 0.9-0.95; women 0.8-0.85), and high risk (men >0.95; women >0.85).

To assess glycemia, we asked the primary health center in each town to conduct a HbA_1c_ test. All blood tests were collected and evaluated by trusted outsourced laboratories that were contracted by the primary health center. All handling procedures were carried out in accordance with national quality and safety standards and under the supervision of the researchers. A value of 8.0% was the standard reference value, as recommended by the American Diabetes Association for individuals aged over 60 years [20]. We also assessed fasting capillary glycemia, which a nursing professional verified at the time of the questionnaire application using an Accu-Check Active glucometer. In this case, 70-110 mg/dL was the standard reference value [20].

During the interview, the dietitian also investigated the clinical and nutritional history of participants, time from T2DM diagnosis, family history of diabetes, and medication intake. The dietitian registered information on the presence of comorbidities, such as hypertension and dyslipidemia, and whether the participant had received any nutritional guidance, engaged in physical activities routinely, smoked, and consumed alcohol. At the end of the interview, the participant received nutritional instructions for better glycemic control and a report generated by the app showing his or her dietary and health status according to the SISVAN guidelines.

### Assessment of the Mobile App With Health Professionals

We also assessed the mobile app with 20 health professionals, including 4 medical doctors, 8 registered nurses, and 8 community health agents (registered nurses and licensed practical nurse) who worked in the primary health centers. Individually, we presented the app and described its functionalities and form of use. We let each professional test and interact with the app and fill out the questionnaire once with dummy data, which we discarded later. After the professionals tested the app, we applied a questionnaire to assess usability. To assess usability, we used an adaptation of the System Usability Scale [21]. To analyze acceptance, we used a questionnaire from the Technology Acceptance Model (TAM) [22]. Last, we asked the health professionals the following 3 descriptive questions: Have you ever used a mobile app in clinical practice? How was your experience with using the Diabetes Food Control 2? What changes do you suggest for this app to make it more useful or applicable to clinical practice? The opinions of professionals from the primary health centers would allow us to understand the potential for implementing the app in clinical practice.

### Data Analysis

Participants’ dietary intakes were summarized and compared by all the demographic, nutritional, biochemical, and lifestyle variables. Intake was classified according to whether it was in line with SISVAN guidelines [16]. We used SPSS 22.0 statistical software (IBM Corp, Armonk, NY) to analyze the quantitative data. Basic quantitative data are described as mean, SD, and median. Categorical data are described by simple frequencies. The associations between dietary intake and demographic, nutritional, biochemical, and lifestyle variables were tested using chi-square tests, considering a 5% significance level for all analyses. For the qualitative data, we assessed the data in groups, following the model proposed by Minayo [23].

## Results

### Participants

Table 1 presents the demographic characteristics. Most of the participants were women, married, with self-reported white skin color, residents of an urban area, with <4 years of education, retired, with family income between 4 and 10 minimum wages, and presenting with a family history of diabetes.

**Table 1:** Demographic characteristics of 154 patients with type 2 diabetes mellitus in primary care in Rio Grande do Sul, Brazil in 2018.

Characteristic			n (%)
**Gender**			
	Female	107 (69.5)	
	Male	47 (30.5)	
**Age (years)**			
	60-69	67 (43.5)	
	70-79	63 (40.9)	
	80-89	22 (14.3)	
	≥90	2 (1.3)	
**Marital status**			
	Single	5 (3.2)	
	Married	101 (65.6)	
	Divorced	5 (3.2)	
	Widow(er)	43 (27.9)	
**Self-reported skin color**			
	White	130 (84.4)	
	Brown	15 (9.7)	
	Black	9 (5.8)	
**Place of residence**			
	Urban area	126 (81.8)	
	Rural area	28 (18.2)	
**Education level**			
	Elementary school	137 (89.0)	
	High school	4 (2.5)	
	Higher education	3 (1.9)	
	Illiterate	10 (6.5)	
**Family income (minimum wages in Brazil)**			
	Between 10 and 20 minimum wages	12 (7.8)	
	Between 4 and 10 minimum wages	70 (45.5)	
	Between 2 and 4 minimum wages	51 (33.1)	
	Up to 2 minimum wages	21 (13.6)	

### Lifestyle, Anthropometric, and Glucose Measurements

Table 2 presents the data on clinical characterization and lifestyle. The majority reported using only oral hypoglycemic agents for T2DM treatment, having dyslipidemia and systemic arterial hypertension, and using medications for hypertension. Most respondents also reported having received some nutritional guidance but not performing nutritional monitoring. Most participants reported having never smoked, not consuming alcohol, and not performing physical activity.

**Table 2:** Clinical characteristics and lifestyle of 154 patients with type 2 diabetes mellitus in primary care in Rio Grande do Sul, Brazil in 2018.

Characteristic			n (%)
Family history of diabetes (yes)			99 (64.3)
**Diabetes medication**			
	Oral hypoglycemic agents	98 (63.6)	
	Insulin only	8 (5.2)	
	Insulin and hypoglycemic agents	35 (22.7)	
	None	13 (8.4)	
Self-reported hypertension (yes)			130 (84.4)
Medication use for hypertension (yes)			76 (97.4)
Self-reported dyslipidemia (yes)			88 (57.1)
Has received nutritional guidance (yes)			89 (57.8)
**Professional providing guidance**			
	Physician	37 (24.0)	
	Dietitian	37 (24.0)	
	Nurse	4 (2.6)	
Nutritional monitoring (yes)			2 (1.3)
**Smoking status**			
	Never smoked	107 (69.5)	
	Former smoker	38 (24.7)	
	Smokes currently	9 (5.8)	
**Frequency of alcohol consumption**			
	Never	121 (78.6)	
	Less than 1 dose per month	22 (14.3)	
	Between 1 and 3 doses per month	7 (4.5)	
	Between 4 and 7 doses per month	4 (2.6)	
Engaged in physical activity (yes)			63 (40.9)

Table 3 presents the characterization of nutritional status. We identified that most participants were obese and had a waist circumference indicating a very high risk of metabolic disorders, a waist-to-hip ratio categorized as high risk, altered capillary glycemia, and altered glycated hemoglobin. As for comorbidities, we identified that 51.9% (80/154) of respondents reported none of the diseases investigated, 31.2% (48/154) with coronary disease, 16.9% (26/154) with diabetic retinopathy, 16.9% (16/154) with depression, 5.8% (9/154) with kidney disease, 1.3% (2/154) with diabetic neuropathy, and 1.3% (2/154) had received a diagnosis of diabetic foot.

**Table 3:** Characterization of nutritional and biochemical status of 154 patients with type 2 diabetes mellitus in primary care in Rio Grande do Sul, Brazil in 2018.

Indicator			Value
Body mass index (kg/m^2^), mean (SD)			31.48 (5.95)
**Body mass index, n (%)**			
	Underweight (<23 kg/m^2^)	7 (4.5)	
	Normal weight (≥23 kg/m^2^ to <28 kg/m^2^)	34 (22.1)	
	Overweight (≥28 kg/m^2^ to <30 kg/m^2^)	25 (16.2)	
	Obesity (≥30 kg/m^2^)	88 (57.1)	
Waist circumference (cm), mean (SD)			108.2 (13.2)
**Waist circumference, n (%)**			
	Low risk (men <94 cm; women <80 cm)	5 (3.2)	
	High risk (men ≥94 cm; women ≥80 cm)	12 (7.8)	
	Very high risk (men ≥102 cm; women ≥88 cm)	137 (89.0)	
Waist-to-hip ratio, mean (SD)			0.999 (0.081)
**Waist-to-hip ratio, n (%)**			
	Low risk (men <0.9; women <0.8)	1 (0.6)	
	Moderate risk (men 0.90:0.95; women 0.80:0.85)	6 (3.9)	
	High risk (men >0.95; women >0.85)	147 (95.5)	
Capillary glycemia (mg/dL), mean (SD)			155 (48)
**Capillary glycemia, n (%)**			
	Normal (70-110 mg/dL)	54 (35.1)	
	Altered (<70 and >110 mg/dL)	100 (64.9)	
**HbA_1c_, mean (SD)**			7.4 (1.4)
	Normal (≤8.0%)	45 (29.2)	
	Altered (>8.0%)	50 (32.5)	
	Not collected	59 (38.3)	

### Dietary Intake

**Figure 2:**
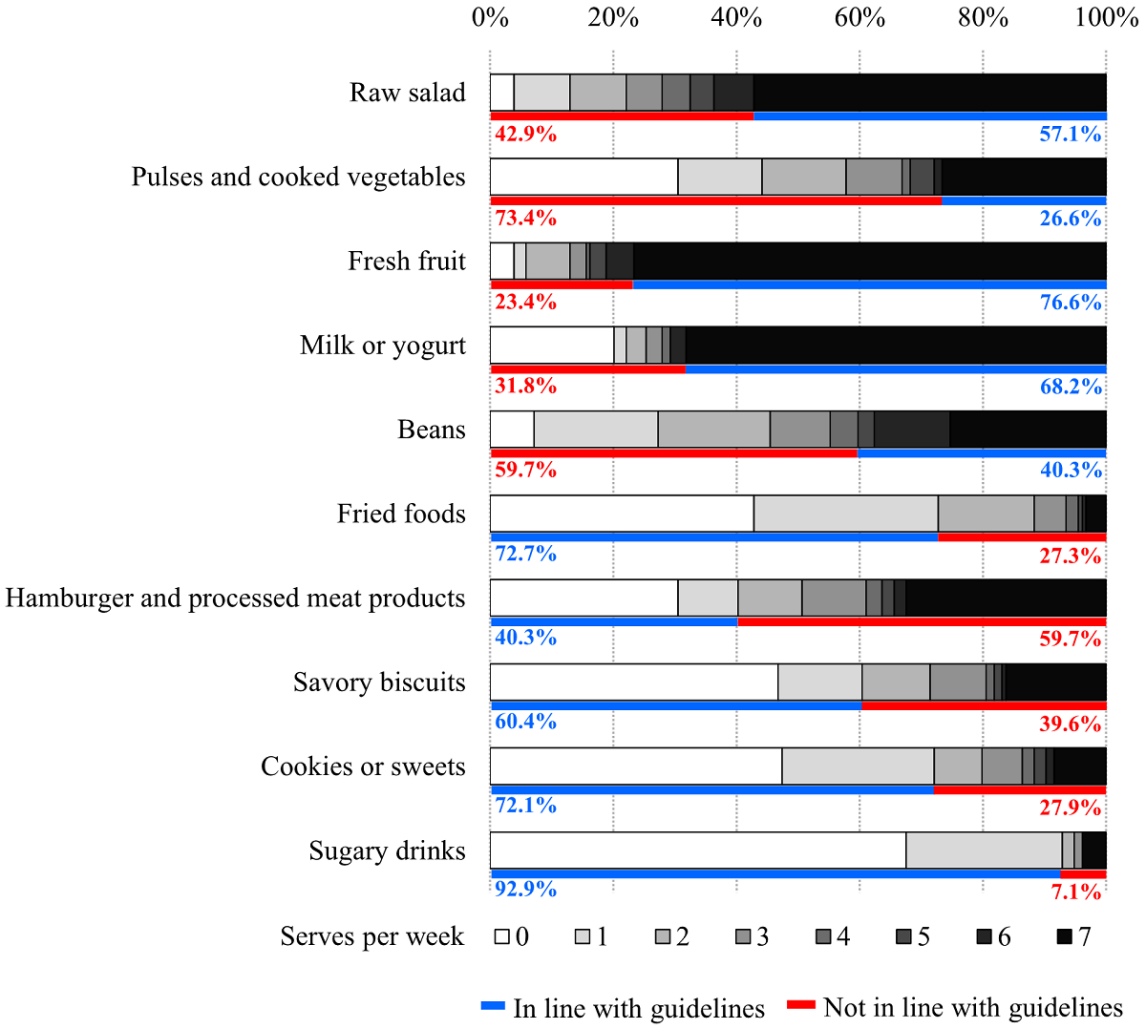
Food frequency according to the number of servings per week and classification according to the Food and Nutrition Surveillance System of Brazil (SISVAN) guidelines from a sample of 154 patients with type 2 diabetes mellitus in primary care in Rio Grande do Sul, Brazil in 2018.

Among the participants, 82.5% (127/154) ate breakfast, 50% (77/154) ate a morning snack, 100% (154/154) ate lunch, 70.1% (108/154) ate dinner, and only 9.1% (14/154) ate supper. Most of them (139/154, 90.3%) described a usual diet in the week prior to the study, 85.1% (131/154) reported not adding salt in prepared food, and 52.6% (81/154) stated using vegetable oil or butter. Most participants had an intake of raw salads, fruits, milk products, fried foods, savory biscuits, cookies or sweets, and sugary drinks in line with recommended guidelines. Meanwhile, the intake of beans, pulses and cooked vegetables, and processed meat products were not in line with recommended guidelines. Figure 2 summarizes the food frequency data for the number of serves per week and the classification according to the SISVAN guidelines. Absolute values are available in Multimedia Appendix 1.

We compared the association between dietary intake with the demographic variables (Multimedia Appendix 2) and with lifestyle, nutritional, and biochemical variables (Multimedia Appendix 3). Statistically, we identified that women had healthier behaviors regarding the intake of fried foods (*χ*^2^_1_=7.963, *P*=.006) and sugary drinks (*χ*^2^_1_=6.127, *P*=.035). As for the place of residence, respondents living in a rural area had a more appropriate intake of cookies and sweets than residents living in an urban area (*χ*^2^_1_=5.035, *P*=.034). Participants without a family history of T2DM had a more appropriate intake of beans (*χ*^2^_1_=15.170, *P*<.001). We detected a more appropriate fruit intake in participants who engaged in physical activity (*χ*^2^_1_=8.955, *P*=.003). We also found that the lower the rate of smoking, the more adequate the intake of raw salad (*χ*^2^_1_=13.034, *P*=.001). Participants with coronary disease reported a more appropriate intake of pulses and cooked vegetables (*χ*^2^_1_=4.223, *P*=.050).

### Health Professionals’ Perceptions About Using the Mobile App

**Figure 3:**
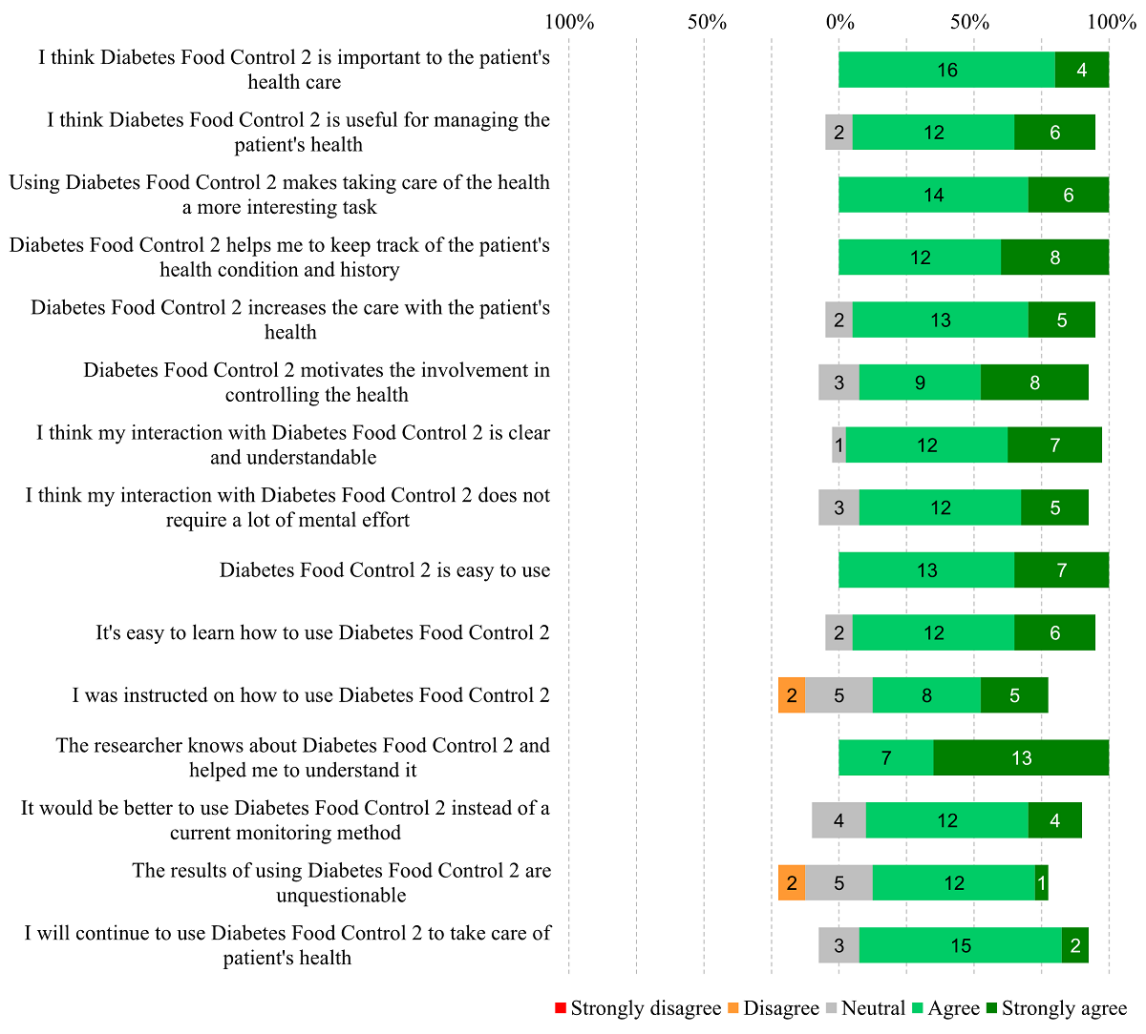
Responses from 20 multidisciplinary, primary care health professionals to the acceptance questionnaire assessing the Diabetes Food control app in Rio Grande do Sul, Brazil in 2018.

The usability assessment with the System Usability Scale generates a score that should be ≥68 points to determine satisfactory results. The assessment of the Diabetes Food Control 2 by health professionals resulted in a mean of 71.75 points, confirming satisfactory usability. Figure 3 presents the responses from health professionals to the TAM questionnaire [22]. The health professionals also answered 3 descriptive questions, depicted in Textbox 1.

**Health professionals’ perceptions about using an app in primary care.**

1. “Have you ever used a mobile app in clinical practice?” No participant had used apps in professional practice.
2. “How did you experience the use of Diabetes Food Control 2?” Among the responses, the professionals mentioned they would have improved knowledge on dietary monitoring of patients, agility and practicality in data collection, higher information reliability, and a more significant scientific basis to guide patients.
3. “What changes do you suggest for this app to make it more useful or applicable to clinical practice” Most professionals said there was no need to change the app because it was already adequate. Among the suggestions for improvements, some professionals mentioned the possibility of having an option to increase the font size in the app (accessibility option), having instructions on how to measure anthropometric variables (eg, where to measure the waist and where to measure the hip), showing the percentage of questionnaire completion, and showing the total completion time.

## Discussion

### Principal Findings

This study assessed various aspects of the nutrition of elderly patients diagnosed with T2DM in the context of primary health in line with SISVAN guidelines, which is a national parameter in Brazil. For data collection, we developed and evaluated a new app based on recommendations from medical associations and literature studies. Interesting insights are presented about the use of the app and the health professionals’ perceptions about its features and the possibility of using it in the context of primary health.

On nutritional assessments, the profile found was similar to surveys performed in the same circumstances involving adult [24] and elderly [25] patients with T2DM. The anthropometric assessment identified that 73.3% of the study population was overweight, which is a rate higher than that presented in similar studies. This stresses the need for greater attention to elderly patients with T2DM assisted in basic health care, especially due to the evident metabolic syndrome and the worsened transition from adulthood to the elderly.

In this study, we identified that men had a greater intake of fried foods than women with a similar dietary behavior, as in the study by Linde et al [26], in which men with higher BMIs consumed more fried foods than women. Similar findings were also reported by Cahill et al [27] and Sayon-Orea et al [28]. Regarding the intake of sugary drinks, we identified that women had a more appropriate intake than men, corroborating other studies [29,30]. As for the place of residence, respondents living in a rural area had a more appropriate intake of cookies and sweets than residents in an urban area, which was similar to the findings of Farrell et al [31] regarding foods and beverages with high sugar content.

The literature has already shown that people who engage in physical activity have better dietary habits [32]. In our study, respondents who reported engaging in physical activity had a more appropriate fruit intake. These data corroborate the studies by Tan et al [33] and Alakaam and Lemacks [34], which identified a positive relationship between physical activity and the intake of fruits and vegetables. We also identified an inversely proportional association between salad intake and smoking. Sebastian et al [35] had similar findings, in which salad intake seemed more prevalent among former smokers and people who had never smoked. Besides, the intake of pulses was significantly higher in participants with cardiovascular disease. Other studies have also associated the intake of fruits and vegetables with a lower prevalence of coronary disease and the intake of fiber with a lower prevalence of acute myocardial infarction [36,37]. Interestingly, our findings also suggest that respondents without a family history of diabetes had a more appropriate intake of beans. Some prior studies discuss the relationship between T2DM and the intake of beans. For instance, Ley et al [38] affirmed that a diet rich in vegetables, legumes, and unrefined grains was associated with a lower risk of T2DM, and for diagnosed patients, the intake of such foods improves glycemic levels and lipid control.

The app we developed has several benefits emphasized by health professionals. Other researchers who used mobile apps in dietary intake assessments cited similar advantages. Rangan et al [39] mentioned a reduction in data entry and coding workload for researchers. Meanwhile, Bucher Della Torre et al [40] reaffirmed that electronic assessment helps to prevent some pitfalls of paper-based food records, such as errors in transcription and difficulties in reading what participants have written. There is a growing interest in using smartphones to collect data on dietary assessment [6], considering mobile technology offers a wide range of suitable options to achieve the benefits that technology can deliver [41-43].

Among the 20 respondents, none had used apps in their professional practice. This suggests the need for implementing the use of technology in public health, considering the potential help and benefits provided to professionals. Health professionals also reported that, by using the app, they could thoroughly monitor patients in the long term, considering that the questionnaire is easy to apply and contains all the questions needed for proper monitoring in the context of primary health. Community health agents, who are professionals who visit patients’ homes, mentioned that the app could be used by them to counsel the general population regarding nutrition. These professionals receive little prior training on nutrition, and the app would provide a scientific basis so they could provide such care to patients.

### Limitations

The physiological aspect of aging was a limitation of the present study. The potential memory loss of the respondents may have affected their answers to retrospective dietary intake. The length of the questionnaire may have also negatively affected the disposition of participants to respond. Another limitation was the impossibility of measuring the HbA_1c_ of all respondents in the laboratory, due to the lack of financial resources. The app was only used one time; therefore, the long-term effectiveness and usefulness should be studied further.

### Conclusions

The dietary intake of the assessed participants is partly appropriate. However, this population was substantially overweight and presented with metabolic syndrome and poor results in the anthropometric and biochemical assessments. Moreover, there was a low intake of pulses and cooked vegetables and beans and a high intake of processed meat products. Mostly, the findings of this study are similar to others in the literature, which reinforces a concerning reality of the health conditions of elderly patients with T2DM. These findings can guide dietary interventions and health education in similar settings. In addition, this study adds to the health informatics literature that a new app can be used in the context of public health in Brazil. The mobile app was useful in the study and well-rated by health professionals in primary health care settings. Its features are encouraging tools for use in future studies. In this study, the app was developed to completely focus on T2DM in primary care settings in Brazil. For application in other settings or for other health conditions, the development method could be adapted or used in a similar way, but considering the specificity of other chronic diseases and according to the specific guidelines available. Future studies can use this study as an example of a location and features where technology is satisfactorily accepted by health care professionals in the first introduction initiative.

## Appendix

Multimedia Appendix 1 Food frequency in absolute number of servings per week.

Multimedia Appendix 2 Intake in line with recommended guidelines categorized by demographic and economic variables.

Multimedia Appendix 3 Intake in line with recommended guidelines categorized by clinical, nutritional, biochemical, and lifestyle variables.

## Abbreviations

CAPES: Coordination and Improvement of Higher Level or Education Personnel
CNPq: National Council for Scientific and Technological Development
FAPERGS: State Funding Agency of Rio Grande do Sul
HbA_1c_: glycated hemoglobin
SISVAN: Food and Nutrition Surveillance System of Brazil
T2DM: type 2 diabetes mellitus
TAM: Technology Acceptance Model
