# Supplementary material for: Dietary Intake and Health Status of Elderly Patients With Type 2 Diabetes Mellitus: Cross-sectional Study Using a Mobile App in Primary Care
Source: JMIR Form Res. 2021 Aug 27;5(8):e27454. doi: 10.2196/27454 (PMC8433854; doi:10.2196/27454)
Supplement: Multimedia Appendix 1 [file formative_v5i8e27454_app1.docx]

|  | **0 serves** | **1 serve** | **2 serves** | **3 serves** | **4 serves** | **5 serves** | **6 serves** | **7 serves** |
| --- | --- | --- | --- | --- | --- | --- | --- | --- |
| **Raw salad** | 6 | 14 | 14 | 9 | 7 | 6 | 10 | 88 |
| **Pulses and cooked vegetables** | 47 | 21 | 21 | 14 | 2 | 6 | 2 | 41 |
| **Fresh fruit** | 6 | 3 | 11 | 4 | 1 | 4 | 7 | 118 |
| **Milk or yogurt** | 31 | 3 | 5 | 4 | 2 | 0 | 4 | 105 |
| **Beans** | 11 | 31 | 28 | 15 | 7 | 4 | 19 | 39 |
| **Fried foods** | 66 | 46 | 24 | 8 | 3 | 1 | 1 | 5 |
| **Hamburger and processed meat products** | 47 | 15 | 16 | 16 | 4 | 3 | 3 | 50 |
| **Savory biscuits** | 72 | 21 | 17 | 14 | 2 | 2 | 1 | 25 |
| **Cookies or sweets** | 73 | 38 | 12 | 10 | 3 | 3 | 2 | 13 |
| **Sugary drinks** | 104 | 39 | 3 | 2 | 0 | 0 | 0 | 6 |

Food frequency in number of servings per week from a sample of 154 patients with Type 2 Diabetes in primary care, Rio Grande do Sul, Brazil, 2018.
