# Supplementary material for: Dietary Intake and Health Status of Elderly Patients With Type 2 Diabetes Mellitus: Cross-sectional Study Using a Mobile App in Primary Care
Source: JMIR Form Res. 2021 Aug 27;5(8):e27454. doi: 10.2196/27454 (PMC8433854; doi:10.2196/27454)
Supplement: Multimedia Appendix 2 [file formative_v5i8e27454_app2.docx]

Intake in line with recommended guidelines categorized by demographic and economic variables (N=154).

|  | **Daily intake** | | | | **Intake ≥ 5 times per week** | **Intake ≤ once a week** | | | | |
| --- | --- | --- | --- | --- | --- | --- | --- | --- | --- | --- |
|  | **Raw salads**  **n (%)** | ***P*ulses and cooked vegetables**  **n (%)** | **Fresh fruits**  **n (%)** | **Milk or yogurt**  **n (%)** | **Beans**  **n (%)** | **Fried foods**  **n (%)** | ***P*rocessed meat products**  **n (%)** | **Savory biscuits**  **n (%)** | **Cookies or sweets**  **n (%)** | **Sugary drinks**  **n (%)** |
| **Gender**  CHI²* | *P=*.596  0.431 | *P=*.79  3.193 | *P*= 0.415  0.693 | *P=*.574  0.539 | *P=*.111  2.719 | ***P=*.006**  **7.963** | *P=*.108  3.085 | *P=*.110  2.729 | *P*=1.000  0.002 | ***P=*.035**  **6.127** |
| Male | 25 (53.2) | 8 (17.0) | 34 (72.3) | 34 (72.3) | 16 (34.0) | 27 (57.4) | 14 (29.8) | 33 (70.2) | 34 (72.3) | 40 (85.1) |
| Female | 63 (58.9) | 33 (30.8) | 84 (7.5) | 71 (66.4) | 23 (21.5) | 84 (79.4) | 48 (44.9) | 60 (56.1) | 77 (72.0) | 103 (96.3) |
|  |  |  |  |  |  |  |  |  |  |  |
| **Years of schooling**  CHI²* | *P=*.693  0.735 | *P=*.884  0.246 | *P=*.586  1.069 | *P=*.573  1.113 | *P=*.260  2.693 | *P=*.521  1.304 | *P=*.999  0.002 | *P=*.163  3.634 | *P=*.370  1.990 | *P=*.933  0.139 |
| ≤ 4 | 56 (56.6) | 27 (27.3) | 75 (75.8) | 65 (65.7) | 27 (27.3) | 69 (69.7) | 40 (40.4) | 64 (64.6) | 68 (68.7) | 92 (92.9) |
| 5 – 8 | 25 (55.6) | 12 (26.7) | 34 (75.6) | 32 (71.1) | 8 (17.8) | 53 (77.8) | 18 (40.0) | 22 (48.9) | 36 (80.0) | 42 (93.3) |
| ≥ 9 | 7 (70.0) | 2 (20.0) | 9 (90.0) | 8 (80.0) | 4 (40.0) | 8 (80.0) | 4 (40.0) | 7 (70.0) | 7 (70.0) | 9 (90.0) |
|  |  |  |  |  |  |  |  |  |  |  |
| ***P*lace of residence**  CHI²* | *P=*.291  1.604 | *P=*.638  0.473 | *P=*.622  0.582 | *P=*.263  1.703 | *P*>.999  0.002 | *P*>.999  0.089 | *P*>.999  0.013 | *P*>.999  0.002 | ***P=*.034**  **5.035** | *P*>.999  0.000 |
| Urban area | 69 (54.8) | 35 (27.8) | 95 (75.4) | 83 (65.9) | 32 (25.4) | 91 (72.2) | 51 (40.5) | 76 (60.3) | 86 (68.3) | 117 (92.9) |
| Rural area | 19 (67.9) | 6 (21.4) | 23 (82.1) | 22 (78.6) | 7 (25.0) | 21 (75.0) | 11 (39.3) | 17 (60.7) | 25 (89.3) | 26 (92.9) |
|  |  |  |  |  |  |  |  |  |  |  |
| **Family income**  CHI²* | *P=*.146  0.386 | *P=*.299  3.671 | *P=*.909  0.544 | *P=*.493  2.404 | *P=*.676  1.528 | *P=*.571  2.007 | *P=*.059  7.428 | *P=*.109  6.049 | *P=*.782  1.081 | *P=*.120  5.834 |
| Between 10 and 20 minimum wages | 8 (38.1) | 5 (23.8) | 17 (81.0) | 13 (61.9) | 6 (28.6) | 17 (81.0) | 14 (66.7) | 9 (42.9) | 14 (66.7) | 21 (100.0) |
| Between 4 and 10 minimum wages | 32 (62.7) | 13 (25.5) | 40 (78.4) | 33 (64.7) | 10 (19.6) | 37 (72.5) | 17 (33.3) | 29 (56.9) | 37 (72.5) | 44 (86.3) |
| Between 2 and 4 minimum wages | 39 (55.7) | 17 (24.3) | 52 (74.3) | 52 (74.3) | 19 (27.1) | 48 (68.6) | 27 (38.6) | 45 (64.3) | 50 (71.4) | 67 (95.7) |
| Up to 2 minimum wages | 9 (75.0) | 6 (50.0) | 9 (75.0) | 7 (58.3) | 4 (33.2) | 10 (83.3) | 4 (33.3) | 10 (83.3) | 10 (83.3) | 11 (91.7) |

CHI²* Chi Square *P*-Values. All degrees of freedom are 1.
