# Supplementary material for: Dietary Intake and Health Status of Elderly Patients With Type 2 Diabetes Mellitus: Cross-sectional Study Using a Mobile App in Primary Care
Source: JMIR Form Res. 2021 Aug 27;5(8):e27454. doi: 10.2196/27454 (PMC8433854; doi:10.2196/27454)
Supplement: Multimedia Appendix 3 [file formative_v5i8e27454_app3.docx]

Intake in line with recommended guidelines categorized by clinical, nutritional, biochemical, and lifestyle variables (N=154).

|  | **Daily intake** | | | | **Intake ≥ 5 times per week** | **Intake ≤ once a week** | | | | |
| --- | --- | --- | --- | --- | --- | --- | --- | --- | --- | --- |
|  | **Raw salads**  **n (%)** | **Pulses and cooked vegetables**  **n (%)** | **Fresh fruits**  **n (%)** | **Milk or yogurt**  **n (%)** | **Beans**  **n (%)** | **Fried foods**  **n (%)** | ***P*rocessed meat products**  **n (%)** | **Savory biscuits**  **n (%)** | **Cookies or sweets**  **n (%)** | **Sugary drinks**  **n (%)** |
| **Family history of diabetes**  CHI²* | *P*=.734  0.236 | *P*=.573  0.391 | *P*=.843  0.116 | *P*>.999  0.033 | ***P*<.001**  **15.170** | *P*=.013  6.987 | *P*=.123  2.774 | *P*=.732  0.174 | *P*=.709  0.259 | *P*=.201  1.830 |
| *P*resence | 58 (58.6) | 28 (28.3) | 75 (75.8) | 67 (67.7) | 15 (15.2) | 79 (79.8) | 35 (35.4) | 61 (61.6) | 70 (70.7) | 94 (94.9) |
| Absence | 30 (54.5) | 13 (23.6) | 43 (78.2) | 38 (69.1) | 24 (43.6) | 33 (60.0) | 27 (49.1) | 32 (58.2) | 41 (74.5) | 49 (89.1) |
|  |  |  |  |  |  |  |  |  |  |  |
| **Self-reported hypertension**  CHI²* | *P*=.373  1.053 | *P*=.212  1.722 | *P*=.600  0.715 | *P*=.817  0.092 | *P*=.617  0.222 | *P*=.464  0.526 | *P*=.366  1.121 | *P*=.824  0.050 | *P*=.809  0.121 | *P*=.379  1.230 |
| *P*resence | 72 (55.4) | 32 (24.6) | 98 (75.4) | 88 (67.7) | 32 (24.6) | 96 (73.8) | 50 (38.5) | 79 (60.8) | 93 (71.5) | 122 (93.8) |
| Absence | 16 (66.7) | 9 (37.5) | 20 (83.3) | 17 (70.8) | 7 (29.2) | 16 (66.7) | 12 (50.0) | 14 (58.3) | 18 (75.0) | 21 (87.5) |
|  |  |  |  |  |  |  |  |  |  |  |
| **Self-reported dyslipidemia**  CHI²* | *P*>.999  0.009 | *P*=.856  0.044 | *P*=.569  0.366 | *P*=.861  0.122 | *P*>.999  0.011 | *P*=.719  0.134 | *P*=.250  1.406 | *P*=.509  0.509 | *P*=.858  0.043 | *P*=.531  0.661 |
| *P*resence | 50 (56.8) | 24 (27.3) | 69 (78.4) | 59 (67.0) | 22 (25.0) | 65 (73.9) | 39 (44.3) | 51 (58.0) | 64 (72.7) | 83 (94.3) |
| Absence | 38 (57.6) | 17 (25.8) | 49 (74.2) | 46 (69.0) | 17 (25.8) | 47 (71.2) | 23 (34.8) | 42 (63.6) | 47 (71.2) | 60 (90.9) |
|  |  |  |  |  |  |  |  |  |  |  |
| **Has received nutritional guidance** CHI²* | *P*=.743  0.142 | *P*=.854  0.066 | *P*=.564  0.484 | *P*=.727  0.213 | *P*=.453  0.853 | *P*=.363  0.998 | *P*=.618  0.371 | *P*=.406  0.840 | *P*=.586  0.453 | *P*>.999  0.051 |
| Yes | 52 (58.4) | 23 (25.8) | 70 (78.7) | 62 (69.7) | 25 (28.1) | 62 (69.7) | 34 (38.2) | 51 (57.3) | 66 (74.2) | 83 (93.3) |
| No | 36 (55.4) | 18 (27.7) | 48 (73.8) | 43 (66.2) | 14 (21.5) | 50 (76.9) | 28 (43.1) | 42 (64.6) | 45 (69.2) | 60 (92.3) |
|  |  |  |  |  |  |  |  |  |  |  |
| ***P*erforms nutritional monitoring**  CHI²* | *P*>.999  0.042 | *P*=.463  0.567 | *P*>.999  0.618 | *P*=.100  4.342 | *P*=.444  0.652 | *P*>.999  0.760 | *P*>.999  0.080 | *P*=.518  1.329 | *P*>.999  0.785 | *P*>.999  0.156 |
| Yes | 1 (50.0) | 1 (50.0) | 2 (100.0) | 0 (0.0) | 1 (50.0) | 2 (100.0) | 1 (50.0) | 2 (100.0) | 2 (100.0) | 2 (100.0) |
| No | 87 (57.2) | 40 (26.3) | 116 (76.3) | 105 (69.1) | 38 (25.0) | 110 (72.4) | 61 (40.1) | 91 (59.9) | 109 (71.7) | 141 (92.8) |
|  |  |  |  |  |  |  |  |  |  |  |
| **Smoking**  CHI²* | ***P*=.001**  **13.034** | *P*=.183  3.392 | *P*=.117  4.299 | *P*=.908  0.193 | *P*=.099  4.621 | *P*=.045  6.182 | *P*=.248  2.787 | *P*=.224  2.995 | *P*=.156  3.716 | *P*=.041  6.367 |
| Never smoked | 71 (66.4) | 33 (30.8) | 87 (81.3) | 72 (67.3) | 25 (23.4) | 84 (78.5) | 41 (38.3) | 60 (56.1) | 75 (70.1) | 103 (96.3) |
| Former smoker | 15 (39.5) | 7 (18.4) | 25 (65.8) | 27 (71.1) | 9 (23.7) | 22 (57.9) | 15 (39.5) | 26 (68.4) | 27 (71.1) | 32 (84.2) |
| Smokes currently | 2 (22.2) | 1 (11.1) | 6 (66.7) | 6 (66.7) | 5 (55.6) | 6 (66.7) | 6 (66.7) | 7 (77.8) | 9 (100.0) | 8 (88.9) |
|  |  |  |  |  |  |  |  |  |  |  |
| **Engages in physical activities** CHI²* | *P*=.049  3.949 | *P*=.268  1.432 | ***P*=.003**  **8.955** | *P*=.488  0.518 | *P*=.457  0.594 | *P*=.466  0.645 | *P*=.739  0.208 | *P*=.320  1.042 | *P*>.999  0.022 | *P*=.760  0.101 |
| Yes | 42 (66.7) | 20 (31.7) | 56 (88.9) | 45 (71.4) | 18 (28.6) | 48 (76.2) | 24 (38.1) | 35 (55.6) | 45 (71.4) | 58 (92.1) |
| No | 46 (50.5) | 21 (23.1) | 62 (68.1) | 60 (65.9) | 21 (23.1) | 64 (70.3) | 38 (41.8) | 58 (63.7) | 66 (72.5) | 85 (93.4) |
|  |  |  |  |  |  |  |  |  |  |  |
| **Self-reported coronary disease**  CHI²* | *P*=.385  0.817 | ***P*=.050**  **4.223** | *P*=.685  0.252 | *P*=.136  2.547 | *P*>.999  0.004 | *P*=.171  2.332 | *P*=.724  0.221 | *P*=.293  1.129 | *P*=.439  0.868 | *P*=.740  0.149 |
| Absence | 58 (54.7) | 23 (21.7) | 80 (75.5) | 68 (64.2) | 27 (25.5) | 81 (76.4) | 44 (41.5) | 67 (63.2) | 74 (69.8) | 99 (93.4) |
| *P*resence | 30 (62.5) | 18 (37.5) | 38 (79.2) | 37 (77.1) | 12 (25.0) | 31 (64.6) | 18 (37.5) | 26 (54.2) | 37 (77.1) | 44 (91.7) |
|  |  |  |  |  |  |  |  |  |  |  |
| **Self-reported diabetic retinopathy** CHI²* | *P*=.084  3.243 | *P*=.055  3.939 | *P*>.999  0.002 | *P*=.360  1.102 | *P*=.228  1.428 | *P*=.637  0.193 | *P*=.381  1.171 | *P*=.827  0.095 | *P*>.999  0.016 | *P*=.213  2.406 |
| Absence | 69 (53.9) | 30 (23.4) | 98 (76.6) | 85 (66.4) | 30 (23.4) | 94 (73.4) | 54 (42.2) | 78 (60.9) | 92 (71.9) | 117 (91.4) |
| *P*resence | 19 (73.1) | 11 (42.3) | 20 (76.9) | 20 (76.9) | 9 (34.6) | 18 (69.2) | 8 (30.8) | 15 (57.7) | 19 (73.1) | 26 (100.0) |
|  |  |  |  |  |  |  |  |  |  |  |
| **Self-reported diabetic neuropathy** CHI²* | *P*=.507  1.520 | *P*>.999  0.735 | *P*=.414  0.802 | *P*=.537  0.309 | *P*>.999  0.687 | *P*=.472  0.528 | *P*=.516  1.366 | *P*=.518  1.329 | *P*=.482  0.491 | *P*>.999  0.156 |
| Absence | 86 (56.6) | 41 (27.0) | 117 (77.0) | 104 (68.4) | 39 (25.7) | 111 (73.0) | 62 (40.8) | 91 (59.9) | 110 (72.4) | 141 (92.8) |
| *P*resence | 2 (100.0) | 0 (0.0) | 1 (50.0) | 1 (50.0) | 0 (0.0) | 1 (50.0) | 0 (0.0) | 2 (100.0) | 1 (50.0) | 2 (100.0) |
|  |  |  |  |  |  |  |  |  |  |  |
| **Self-reported kidney disease** CHI²* | *P*>.999  0.010 | *P*=.446  1.177 | *P*=.033  5.526 | *P*>.999  0.010 | *P*>.999  0.049 | *P*=.257  1.421 | *P*=.741  0.191 | *P*>.999  0.157 | *P*=.710  0.139 | *P*=.496  0.227 |
| Absence | 83 (57.2) | 40 (27.16) | 114 (78.6) | 99 (68.3) | 37 (25.5) | 107 (73.8) | 59 (40.7) | 78 (60.0) | 105 (72.4) | 135 (93.1) |
| *P*resence | 5 (55.6) | 1 (11.1) | 4 (44.4) | 6 (66.7) | 2 (22.2) | 5 (55.6) | 3 (33.3) | 6 (66.7) | 6 (66.7) | 8 (88.9) |
|  |  |  |  |  |  |  |  |  |  |  |
| **Self-reported diabetic foot** CHI²* | *P*=.507  1.520 | *P*=.463  0.567 | *P*=.414  0.802 | *P*>.999  0.946 | *P*=.444  0.652 | *P*>.999  0.760 | *P*=.161  3.007 | *P*>.999  0.091 | *P*>.999  0.785 | *P*=.138  5.611 |
| Absence | 86 (56.6) | 40 (26.3) | 117 (77.0) | 103 (67.8) | 38 (25.0) | 110 (72.4) | 60 (39.5) | 92 (60.5) | 109 (71.7) | 142 (93.4) |
| *P*resence | 2 (100.0) | 1 (50.0) | 1 (50.0) | 2 (100.0) | 1 (50.0) | 2 (100.0) | 2 (100.0) | 1 (50.0) | 2 (100.0) | 1 (50.0) |
|  |  |  |  |  |  |  |  |  |  |  |
| **Self-reported depression** CHI²* | *P*>.999  0.004 | *P*=.149  2.244 | *P*=.800  0.300 | *P*=.649  0.346 | *P*>.999  0.084 | *P*=.469  1.020 | *P*=.027  5.889 | *P*=.512  0.560 | *P*=.343  1.174 | *P*=.213  2.406 |
| Absence | 73 (57.0) | 31 (24.2) | 97 (75.8) | 19 (73.1) | 33 (25.8) | 91 (71.1) | 46 (35.9) | 79 (61.7) | 90 (70.3) | 117 (91.4) |
| *P*resence | 15 (57.7) | 10 (38.5) | 21 (80.8) | 86 (67.2) | 6 (23.1) | 21 (80.8) | 16 (61.5) | 14 (53.8) | 21 (80.8) | 26 (100.0) |
|  |  |  |  |  |  |  |  |  |  |  |
| **Body mass index**  CHI²* | *P*=.819  0.928 | *P*=.489  2.426 | *P*=.270  3.926 | *P*=.321  3.499 | *P*=.733  1.284 | *P*=.999  0.024 | *P*=.609  1.828 | *P*=.058  7.502 | *P*=.374  3.114 | *P*=.042  8.178 |
| Underweight | 4 (57.1) | 1 (14.3) | 7 (100.0) | 3 (42.9) | 3 (42.9) | 5 (71.4) | 3 (42.9) | 1 (14.3) | 6 (85.7) | 5 (71.4) |
| Normal weight | 17 (50.0) | 12 (35.3) | 28 (82.4) | 25 (73.5) | 9 (26.5) | 25 (73.5) | 17 (50.0) | 19 (55.9) | 24 (70.6) | 33 (97.1) |
| Overweight | 15 (60.0) | 5 (20.0) | 17 (68.0) | 15 (60.0) | 6 (24.0) | 18 (72.0) | 9 (36.0) | 17 (68.0) | 21 (84.0) | 25 (100.0) |
| Obesity | 52 (59.1) | 23 (26.1) | 66 (75.0) | 62 (70.5) | 21 (23.9) | 64 (72.7) | 33 (37.5) | 56 (63.6) | 60 (68.2) | 80 (90.9) |
|  |  |  |  |  |  |  |  |  |  |  |
| **Waist circumference**  CHI²* | *P*=.775  0.510 | *P*=.933  0.139 | *P*=.295  2.440 | *P*=.381  1.928 | *P*=.145  3.866 | *P*=.027  7.245 | *P*=.550  1.196 | *P*=.384  1.916 | *P*=.566  1.138 | *P*=.512  1.340 |
| Low risk | 3 (60.0) | 1 (20.0) | 4 (80.0) | 2 (40.0) | 3 (60.0) | 1 (20.0) | 1 (20.0) | 3 (60.0) | 3 (60.0) | 4 (80.0) |
| High risk | 8 (66.7) | 3 (25.0) | 7 (58.3) | 8 (66.7) | 4 (33.3) | 9 (75.0) | 4 (33.3) | 5 (41.7) | 10 (83.3) | 11 (91.7) |
| Very high risk | 77 (56.2) | 37 (27.0) | 107 (78.1) | 95 (69.3) | 32 (23.4) | 102 (74.5) | 57 (41.6) | 85 (62.0) | 98 (71.5) | 128 (93.4) |
|  |  |  |  |  |  |  |  |  |  |  |
| **Waist-hip ratio**  CHI²* | *P*=.646  0.875 | *P*=.707  0.693 | *P*=.725  0.642 | *P*=.082  4.990 | *P*=.082  5.005 | *P*=.074  5.219 | *P*=.666  0.813 | *P*=.356  2.066 | *P*=.789  0.475 | *P*=.754  0.564 |
| Low risk | 1 (100.0) | 0 (0.0) | 1 (100.0) | 0 (0.0) | 1 (100.0) | 1 (100.0) | 0 (0.0) | 1 (100.0) | 1 (100.0) | 1 (100.0) |
| Moderate risk | 3 (50.0) | 1 (16.7) | 4 (66.7) | 6 (100.0) | 0 (0.0) | 2 (33.3) | 2 (33.3) | 5 (83.3) | 4 (66.7) | 6 (100.0) |
| High risk | 84 (57.1) | 40 (27.2) | 113 (76.9) | 99 (67.3) | 38 (25.9) | 109 (74.1) | 60 (40.8) | 87 (59.2) | 106 (72.1) | 136 (92.5) |
|  |  |  |  |  |  |  |  |  |  |  |
| **Capillary glycemia**  CHI²* | *P*=.735  0.152 | *P*=.570  0.385 | *P*=.690  0.302 | *P*=.856  0.088 | *P*=.565  0.423 | *P*=.449  0.743 | *P*=.864  0.065 | *P*=.730  0.230 | *P*=.347  1.210 | *P*=.518  0.562 |
| Normal | 32 (59.3) | 16 (29.6) | 40 (74.1) | 36 (66.7) | 12 (22.2) | 37 (68.5) | 21 (38.9) | 34 (63.0) | 36 (66.7) | 49 (90.7) |
| Altered | 56 (56.0) | 25 (25.0) | 78 (78.0) | 69 (69.0) | 27 (27.0) | 75 (75.0) | 41 (41.0) | 59 (59.0) | 75 (75.0) | 94 (94.0) |
|  |  |  |  |  |  |  |  |  |  |  |
| **HbA1c**  CHI²* | *P*=.382  1.100 | *P*=.328  1.421 | *P*=.683  0.354 | *P*=.360  0.926 | *P*>.999  0.026 | *P*=.699  0.281 | *P*=.861  0.066 | *P*=.289  1.454 | *P*=.568  0.421 | *P*=.175  2.821 |
| Normal | 57 (54.3) | 31 (29.5) | 79 (75.2) | 69 (65.7) | 27 (25.7) | 75 (71.4) | 43 (41.0) | 60 (57.1) | 74 (70.5) | 95 (90.5) |
| Altered | 31 (63.3) | 10 (20.4) | 39 (79.6) | 36 (73.5) | 12 (24.5) | 37 (75.5) | 19 (38.8) | 33 (67.3) | 37 (75.5) | 48 (98.0) |

CHI²* Chi Square *P*-Values. All degrees of freedom are 1.
